# *Aloe vera* gel relieves cadmium triggered hepatic injury via antioxidative, anti-inflammatory, and anti-apoptotic routes

**DOI:** 10.1007/s12011-024-04141-4

**Published:** 2024-03-12

**Authors:** Rasha E. Hassan, Eman M. Saleh, Germine M. Hamdy

**Affiliations:** Biochemistry Department, Faculty of Science, Ain Shams University, Abbassia, Cairo, 11566 Egypt

**Keywords:** Cadmium, *Aloe vera* gel, Hepatotoxicity, Oxidative stress, Inflammation, Apoptosis

## Abstract

*Aloe vera* (*AV*) gel extracted from fresh *AV* leaves was chosen in this study to evaluate its antioxidant, anti-inflammatory, and antiapoptotic activities against cadmium (Cd) -induced liver injury. Forty Wistar male adult rats were equally divided into four groups. Group I (standard control) ingested with 2.5 ml/kg b.w. of physiological saline. Group II (Cd-intoxicated) received 3 mg/kg b.w./day of CdCl_2_ dissolved in saline. Group III (*AV*) received 200 mg/kg b.w./day of *AV* gel dissolved in saline. Group IV (Cd+*AV*) ingested with 200 mg/kg b.w./day of *AV* gel solution along with 3 mg/kg b.w. CdCl_2_. All groups were ingested orally by gavage for 3 consecutive weeks. Paraoxonase-1 (PON-1) and HSP70 were measured in serum. The deposited Cd level, nitric oxide content, lipid peroxidation, collagen-1 (COL-1), and metalloproteinase-9 (MMP-9) levels were all determined in liver tissue homogenates. Gene expression of NF-κB and IL-6, Bax, and Bcl2, as well as immunohistochemistry analysis of activated caspase-3, was performed. Results showed that ingestion of *AV* gel greatly relieved all oxidative stress due to Cd exposure, modulated the NF-κB, IL-6, Bax, and Bcl2 expression levels, and improved the apoptotic state. In conclusion, *AV* gel confirmed its potential ameliorating effect against liver injury induced due to Cd exposure.

## Introduction

Cadmium (Cd) is one of the highly toxic heavy metal pollutants that have received considerable attention due to its toxicity, long-term persistence, and ability to affect almost all vital organs in animal systems [1]. It is classified as “Group 1” carcinogenic to humans by the International Agency for Research on Cancer (IARC) [2]. Cd is a common harmful metal in the environment that is readily absorbed by vegetables and grains grown in contaminated soil. Cd is also released into the aquatic environment from agricultural, industrial, and urban sewage discharged into a river or the sea, as well as natural sources such as rocks and soils. As a result, individuals can be exposed to Cd through food consumption, drinking water, and accidental intake of soil sullied by this toxic element [3]. Many aquatic organisms have a high Cd enrichment capacity, and many fish in contaminated areas are seriously over standard. Thus, oral ingestion of Cd is ultimately the primary route of Cd exposure [4].

The liver is a primary Cd target organ, and it is extremely sensitive to both acute and chronic Cd exposures [5]. Because it is primarily accumulated in the liver, Cd has emerged as a significant experimental hepatotoxicant, with acute exposure to toxic doses causing liver damage, and hence apoptosis [6]. Cd-induced liver injury relies not only on the reactive oxygen species (ROS) generation but also on the depletion of antioxidant levels, resulting in an oxidant/antioxidant imbalance and oxidative stress [7]. Primary injury to hepatocytes is induced by Cd binding to sulfhydryl groups, and their inactivation leads to mitochondrial dysfunction, mitochondrial permeability transition, and oxidative stress. Secondary injury to the liver occurs as a result of inflammation caused by Kupffer cells activation. Infiltrating neutrophils, macrophages, as well as resident cells (hepatocytes, endothelial cells, and stellate cells) synthesize and release various cytokines, chemokines, and other proinflammatory mediators, exacerbating the initial Cd-induced injury [8]. Cadmium’s hepatotoxicity is primarily caused by its interaction with important subcellular locations such as the cytosol, mitochondria, peroxisomes, as well as microsomes, which results in excessive free radical production. In turn, these free radicals can produce oxidative stress, which causes oxidative damage to biomolecules including lipids, proteins, and DNA, leading to a range of clinical diseases. Importantly, peroxidation of the bio membrane of lipids is an early indicator of Cd-induced oxidative damage [9]. As a result, studies on natural-based protective agents for Cd-induced hepatotoxicity and apoptosis have been conducted.

Herbal medicines have long been recognized as the most common alternative remedies and protective agents. *Aloe vera* (*AV*) or *Aloe barbadensis Miller* is a cactus-like plant that belongs to the family *Liliaceae* and genus *Aloe. AV*, which has medicinal properties, has been used in traditional medicine for over 2000 years and has a long history of acceptance as a herbal remedy [10]. It has remained a significant element in many cultures’ conventional medicine, including Egypt, China, India, and Japan. Several studies have shown that *AV* species have antioxidant and anti-inflammatory activities [11]. Aloe is a perennial succulent plant with stemless large, thick, and fleshy leaves. Its leaves contain yellow latex, which is referred to as aloe juice or sap which has a bitter taste and is primarily used for its laxative effect. The innermost portion of the leaf, the leaf pulp, is made up of parenchymal cells that contain the gel. Nowadays, the processing of gel derived from the plant’s leaf pulp is widely used for medicinal and cosmetic purposes, and it has become a large global industry.

*AV* gel contains a plethora of biologically active substances, as well as numerous enzymes involved in pain and inflammation relief [12]. As a consequence, *AV* gel has been widely promoted and used to treat a variety of inflammatory digestive and skin diseases, including inflammatory bowel disease [13]. However, the mechanism supporting the protective role of *AV* gel against Cd-induced hepatotoxicity and apoptosis has not been well illustrated. So, this study was intended to assess the hepatoprotective mechanism of *AV* gel against Cd-induced liver damage and apoptosis in rats.

## Materials and Methods

### Chemicals

Cadmium chloride (CdCl_2_), powder (≥99.5%) (product No. 202908), was bought from Sigma-Aldrich, St. Louis, MO, USA. Commercial ELISA kits utilized in this study were provided from Cloud-Clone Corp. (USA) and Uscn Life Science Inc. (China). All other chemicals involved in the current study were of high analytical grade.

### Preparation of *Aloe vera* (*AV*) Gel Extract

The whole *AV* plant was obtained from the agriculture ministry at Giza, and extraction of *AV* gel was conducted according to the method previously described by [14]. The home-made whole gel extract was prepared from mature, healthy, and fresh leaves of *AV*, with an approximate length of 0.75–0.90 m. The leaves were washed with fresh water and sliced to separate the gel by scratching. The thick epidermis was selectively removed. The inner colorless, mucilaginous parenchyma pulp was removed, homogenized in an electric homogenizer (Mechanika Precyzyjna Warszawa Universal Laboratory Aid type homogenizer MPW-309, Poland), and centrifuged at 6400 g at 4 °C for 15 min to remove the fibers. The resultant supernatant was lyophilized immediately. The lyophilized sample was further extracted with 95% ethanol. The filtrate was collected and evaporated to dryness under a reduced pressure of 250 mmHg in a rotary evaporator. The residue was stored in dry sterilized containers at 4 °C until further use. Each time, a known amount of solvent-free extract was resuspended in sterilized saline and administered intragastrically.

### Experimental Animals

The experimental protocol, including animal handling and the described procedures, was carried out in accordance with the guidelines issued by the Faculty of Science’s Ethical Committee for Animal Studies, with the approval of the local institutional animal ethics committee (approval code: ASU-SCI/BIOC/2023/2/2). The current study was conducted on forty adults male Wistar rats (10–12 weeks old) weighing 175–200 g, provided from the breeding section of the Medical Research Center, Faculty of Medicine, Ain Shams University, Cairo, Egypt. Animals were acclimatized for 1 week before starting the experiment with free access to distilled water and standard rodent pellets obtained from the Agricultural Industrial Integration Company, Giza, Egypt. The required conditions were set at 23 ± 2 °C for temperature, 55 ± 5% relative humidity, and a light-dark cycle of 12:12 h.

### Experimental Protocol

Following the acclimatization phase, rats were randomly separated into four groups of ten animals each: Group I (standard normal control; NC): rats received physiological saline (2.5 ml/kg b.w.) orally for 3 consecutive weeks. Group II (Cd-ingested group): animals received a dose of 3 mg/kg b.w./day from CdCl_2_ dissolved in saline, orally for 3 consecutive weeks, as described by [15]. Group III (*Aloe vera* group; *AV*): rats were orally ingested with a dose of 200 mg/kg b.w./day of *AV* gel (dissolved in saline) for 3 weeks, after Hassanshahi et al. (2020) [16]. Group IV (Cd*+AV*): animals in this group received orally 200 mg/kg b.w./day of *AV* gel solution along with 3 mg/kg b.w. CdCl_2_.

### Blood and Tissue Sampling

At the end of the experimental period, all groups were fasted overnight and anesthetized i.p. with sodium pentobarbital (50 mg/kg b.w.). A cardiac piercing procedure was used to collect blood samples, which were then allowed to clot for 30 min at room temperature before being centrifuged for 15 min at 3000 rpm. Collected sera were separated into aliquots, kept at −20 °C for the assessment of PON-1, HSP70, as well as apoptotic and anti-apoptotic markers. Immediately after sacrifice, liver tissues were perfused, dissected out, divided into three parts; the first part (~ 0.1 g) was placed in sterilized microfuge tube and kept at −80 °C for qRT-PCR, the second part was rinsed in isotonic saline solution, blotted dry with filter paper, weighed, and homogenized with ice-cold PBS (0.01mol/L, pH 7.0-7.2) for Cd deposition, nitric oxide content and lipid peroxidation determinations, as well as liver fibrosis investigations. The third part was used for immunohistochemical analysis.

### Determination of Cd Levels in Liver Homogenates

The accumulated Cd in liver homogenates of rats after oral exposure to 3 mg/kg/day for 3 consecutive weeks was evaluated by the atomic absorption technique according to the previous method of **[**17**]**.

### Oxidative Stress Markers

#### Determination of Nitric Oxide Content

Miranda et al. [18] approach was used to measure nitric oxide in the liver tissue homogenates of all studied groups.

#### Lipid Peroxidation Determination

The amount of thiobarbituric acid reactive substances (TBARS) obtained by the reaction of thiobarbituric acid (TBA) with malondialdehyde (MDA) in the liver tissue homogenates was used to calculate lipid peroxidation. MDA equivalents were used to express the levels of lipid peroxidation products [19].

#### Paraoxonase-1 and HSP70 Markers

The concentrations of paraoxonase-1 (PON-1; Cat. No. SEA243Ra) and heat shock protein70 (HSP70; Cat. No. E90873Ra) were measured in serum using the sandwich ELISA assay kits purchased from Cloud-Clone Corp. (Katy, USA) and Uscn Life Science Inc., Wuhan, China, respectively.

### Inflammatory and Apoptotic Markers Assessment

#### NF-κB p65, IL-6, Bax, and Bcl2 mRNA Expression Levels

The gene expression of the nuclear factor-kappa B (NF-κB) p65 subunits, IL-6, Bax, and Bcl2 was assessed in liver tissue homogenates of all studied groups to evaluate the cadmium’s ability to induced inflammation and apoptosis, as well as the protective impact of *AV* gel against this cascade of injury. Total RNA was extracted from liver tissues using the BIOLINE TRI sure TM kit (Cat. no. BIO-38032), as directed by the manufacturer. Then, according to the manufacturer’s instructions, 1 μg of the extracted RNA was utilized for cDNA synthesis using the BIOLINE Sensi Fast TM cDNA synthesis kit (Cat. no. BIO-65053). The relative expression levels of mRNA encoding NF-κB, IL-6, Bax, Bcl2, and β-actin were measured using the Sensi FAST TM SYBR® No-ROX kit (2X) (Cat. no. BIO-98005) according to manufacturer’s instructions. Results were computerized using Stratagene (Mx 3000PTM) machine. The sequence of the primers used was: 5′-TGCAGGCTCCTGTGCGAGTG-3′ (F) and 5′-TCCGGTGGCGATCGTCTGTGT-3′ (R) for NF-κB p65, 5′-CCAGCCAGTTGCCTTCTTGGGA-3′ (F) and 5′-GGCATAGCACACTAGGTTTGCCGA-3′ (R) for IL-6, 5′-CGGCGAATTGGAGATGAACTGG-3′ (F) and 5′-CTAGCAAAGTAGAAGAGGGCAACC-3′ (R) for Bax, 5′-ATCGCTCTGTGGATGACTGAGTAC-3′ (F) and 5′-AGAGACAGCCAGGAGAAATCAAAC-3′ (R) for Bcl2, and 5′-AGCCATGTACGTAGCCATCC-3′ (F) and 5′-CTCTCAGCTGTGGTGGTGAA-3′ (R) for ß-actin. The expression level of the target genes was normalized to the selected housekeeping one (β-actin) and presented as fold change relative to the standard control group.

#### Evaluation of Liver Collagen-1 and MMP-9

Liver extracellular matrix collagen-1 (COL-1a1; Cat. No. SEA350Ra) content and MMP-9 (Gelatinase B; Cat. No. SEA553Ra) concentrations were determined in the liver tissue homogenates using the ELISA kits purchased from Cloud-Clone Corp. (Katy, USA), according to the manufacturer’s guidelines to explore the impact of Cd and *AV* gel ingestion on liver microenvironment.

### Immunohistochemical Analysis

Immunohistochemical analysis of caspase-3 was performed to confirm the impact of *AV* gel on the Cd-induced apoptosis. Tissue sections (5 μm thick) were treated with 0.03% H_2_O_2_ for 20 min and incubated with polyclonal rabbit antibody anti-cleaved caspase-3 (primary antibody), diluted 1:100 overnight at 4 °C. Slides were then washed and incubated with the secondary antibody (biotinylated anti-rabbit IgG) for 30 min. Streptavidin–biotin or avidin–biotin peroxidase (ABC/HRP) was applied for 10 min at room temperature. The reaction product was visualized by incubating the slides in a solution of 0.02% 3,3-diaminobenzidine tetrahydrochloride (DAB) containing 0.01% H_2_O_2_ for 10 min. Counter staining was performed using Mayer’s hematoxylin, and the slides were visualized under a light microscope using Olympus® digital camera installed on Olympus® light microscope (Olympus, Japan), using 40 × objective [20].

### Statistical Analysis

Statistical analysis of obtained data was carried out and analyzed by Statistical Package for Social Science (SPSS) version 23.0 for Windows (SPSS® Chicago, IL, USA) software program. For quantitative parametric results, representative data was expressed as the mean ± standard error (SE) of replicate determinations. Analysis of variance (ANOVA) was used to test for differences in variable means across groups, and the results were interpreted as follows: *p* ≤ 0.05: significant results, *p* ≤ 0.01 and *p* ≤ 0.001: highly significant results.

## Results

### Deposition of Cd in Liver Tissues of All Studied Groups

**Fig. 1:**
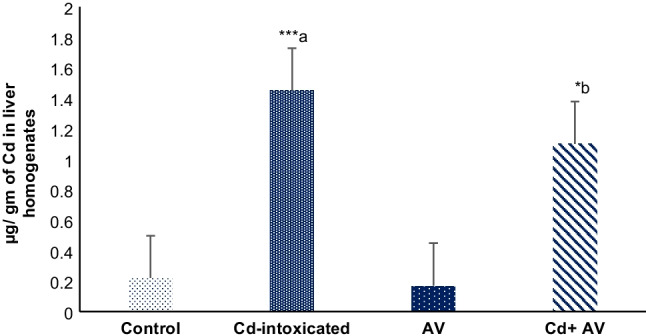
Concentration of Cd in liver homogenate tissues of the studied groups. Data are presented as mean ± SE (*n* = 10). ^***a^*p* ≤ 0.001 and ^*b^*p* ≤ 0.05 in comparison to healthy normal control and Cd-ingested groups, respectively

Figure 1 showed a highly significant (*p* ≤ 0.001) elevation in the measures of Cd scattered in the hepatic tissues of Cd-poisoned group, versus the control one. This implies Cd deposition in the liver following oral exposure to the chosen dose. Notably, these accumulated levels were considerably (*p* ≤ 0.05) reduced in the hepatic tissues of rats treated with Cd + *AV,* when compared to Cd-ingested rats. Meanwhile, no changes in the Cd levels were found in the *AV* gel group when compared to the rats of the standard control group.

### Ameliorative Effect of *AV* Gel Against Cd-Induced Oxidative Stress

#### NO Content Determination

**Fig. 2:**
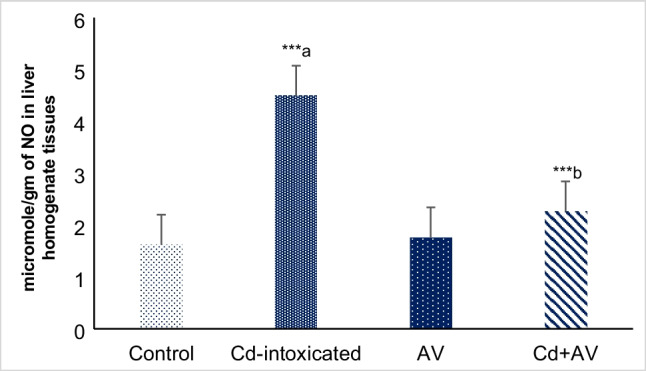
Impact of Cd and *AV* gel on hepatic NO concentration. Values are presented as mean ± SE (*n* = 10). ^***a^*p* ≤ 0.001 and ^***b^*p* ≤ 0.001 in comparison to healthy control and Cd-ingested groups, respectively

Figure 2 Depicts the dramatic significant increase (^***a^*p* ≤ 0.001) in the NO concentration in the liver tissue of Cd-ingested group, versus the control one. On contrary, the administration of *AV* gel markedly reduced (^***b^*p* ≤ 0.001) these levels in group IV, compared to the Cd-intoxicated animals. No obvious changes were recorded in the concentrations of NO among *AV* gel ingested animals and their normal untreated counterparts.

#### PON-1 and HSP70 Levels

**Fig. 3:**
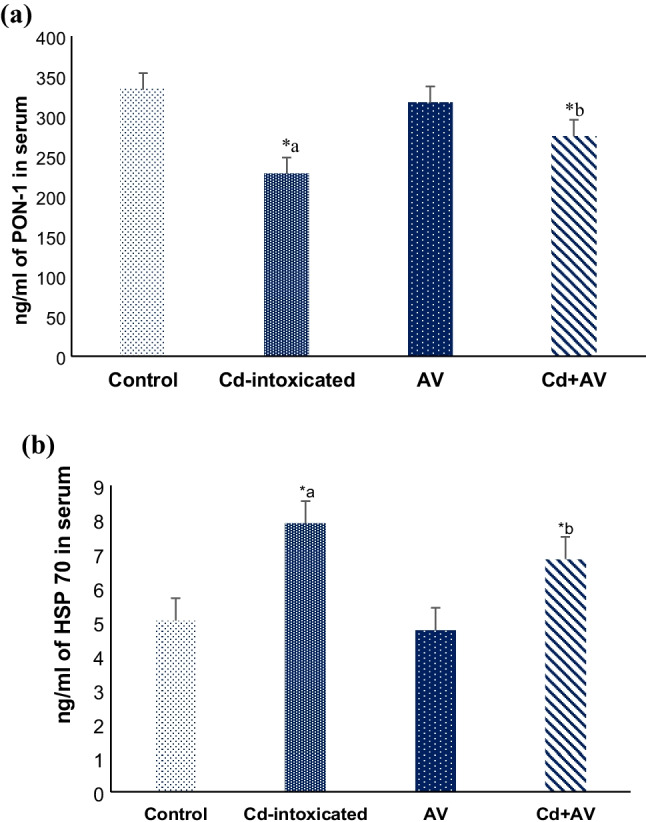
Effects of Cd exposure and *AV* gel ingestion on PON-1 (**a**) and HSP70 (**b**) concentrations. Values are presented as mean ± SE (*n*=10). ^*a^*p* ≤ 0.05 compared with healthy control group, and ^*b^*p* ≤ 0.05 compared with Cd-ingested group

The effect of *AV* gel on Cd-induced hepatotoxicity was evaluated by measuring PON-1 and HSP70 concentrations in serum samples from experimental rats. Notably, a moderate significant decrease in PON-1 concentration concomitant with a considerable elevation (*p* ≤ 0.05) in the HSP70 levels in the Cd-ingested group, versus the healthy normal animals. Instead, oral ingestion of *AV* gel along with Cd significantly counteracted these effects and almost normalized the concentrations of PON-1 and HSP70. Obviously, there was no significant difference in the investigated parameters between the *AV* gel group and the standard control one (Fig. 3a, b).

#### Lipid Peroxidation

**Fig. 4:**
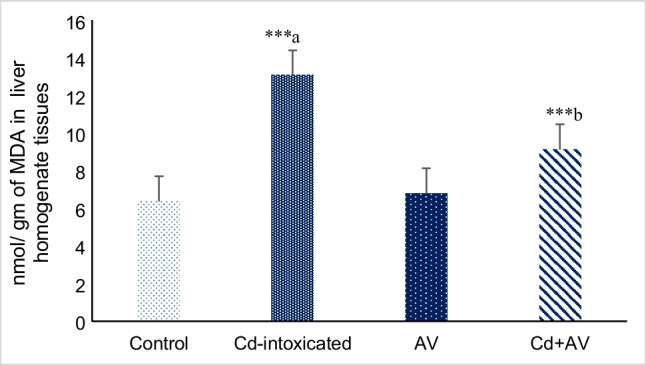
Effects of *AV* gel and Cd on MDA concentrations in liver homogenate tissues of rats. Data are presented as mean ± SE (*n* = 10). ^***a^*p* ≤ 0.001 and ^***b^*p* ≤ 0.001 in comparison to healthy normal control and Cd-ingested groups, respectively

In order to evaluate the protective impact of *AV* gel against Cd-induced oxidative stress in studied rats, the levels of MDA were determined. Figure 4 depicted that the MDA level was significantly increased (*p* < 0.001) among Cd-ingested rats, as comparison to the untreated group. However, co-administration of rats with *AV* gel, along with Cd, significantly suppressed this elevation. Meanwhile, no significant change in the serum levels of MDA was detected among rats ingested with *AV* gel alone (groups III). These results demonstrate the antioxidant and protective effects of our *AV* gel agent against Cd-induced oxidative stress and its ability to enhance the cellular antioxidant defenses.

### Anti-Inflammatory Markers by Gene Expression (NF-κB and IL-6)

**Fig. 5:**
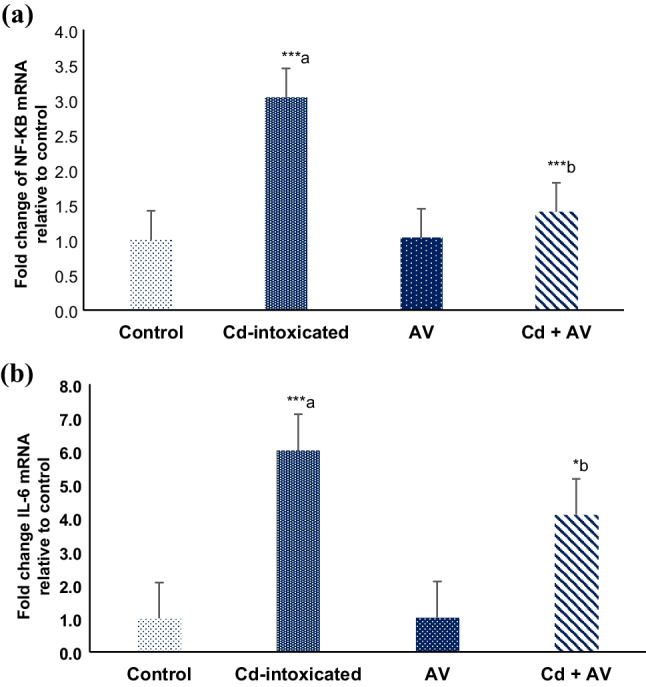
Modulatory effects of *AV* gel against Cd-induced changes in mRNA expression level of NF-kB (**a**) and IL-6 (**b**). The data are presented as mean ± SE (*n* = 10). ^***a^*p* ≤ 0.001 in comparison to the healthy control group. ^*b^*p* ≤ 0.05 and ^***b^*p* ≤ 0.001 in comparison to Cd-ingested group

Results showed a highly significant upregulation (*p* ≤ 0.001) in the mRNA expression level of hepatic NF-KB (3-fold) (Fig. 5a), IL-6 (sixfold) (Fig. 5b), following Cd ingestion in group II, compared to the standard control group. However, *AV* gel co-administration with Cd markedly ameliorated the expression level of inflammatory markers: NF-KB and IL-6 compared to Cd-ingested group.

### Apoptotic Status in Hepatic Tissues

**Fig. 6:**
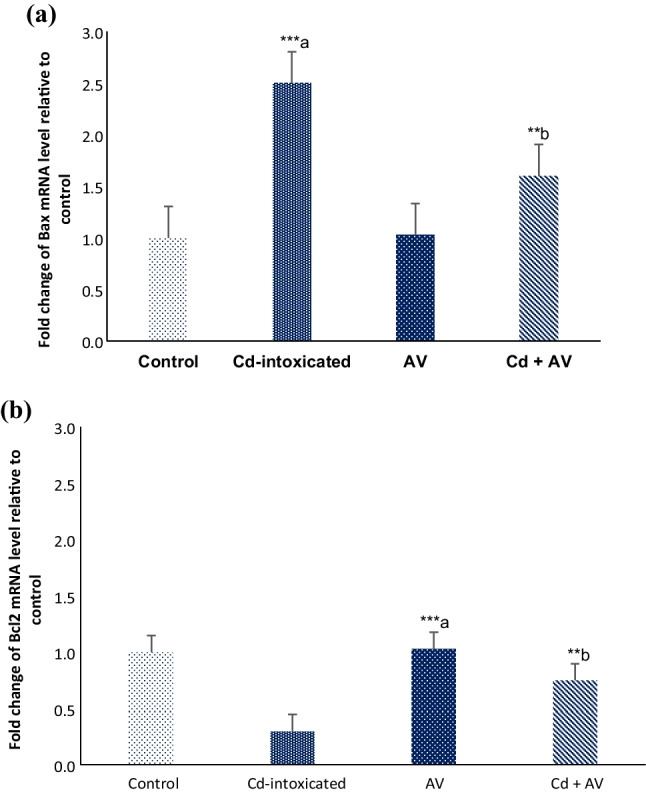
Antiapoptotic effects of *AV* gel against Cd-induced apoptosis in hepatic tissues. mRNA expression level of Bax (**a**) and Bcl2 (**b**). The data are presented as mean ± SE (*n* = 10). ^***a^*p* ≤ 0.001 in comparison to the healthy control group. ^**b^*p* ≤ 0.005 in comparison to Cd-ingested group

The current results revealed a significant increase (*p* ≤ 0.001) in the expression of the pro-apoptotic factor, Bax, and a significant decrease (*p* ≤ 0.001) in the anti-apoptotic Bcl-2 mRNA expression levels following Cd exposure. On the other hand, coadministration of *AV* gel along with Cd mitigates this dramatic apoptotic status in the hepatic tissues as noted by the significant downregulation (*p* ≤ 0.005) in the Bax mRNA expression with concomitant upregulation (*p* ≤ 0.005) in the Bcl2 expression levels (Fig. 6a, b).

### Determination of Caspase-3 Expression by Immunohistochemistry

**Fig. 7:**
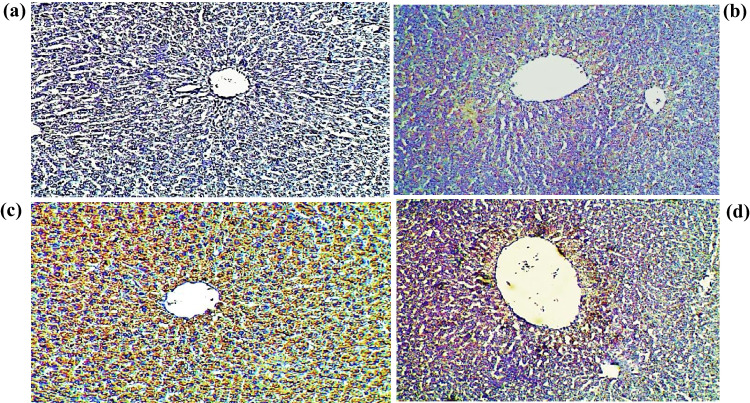
Immunohistochemical staining for cleaved caspase-3 protein in liver sections of studied groups. **a**: section of liver from normal control rats showing negative staining, indicating no expression of protein (100×). **b**: section of liver from a Cd-ingested group with strong positivity for cleaved-caspase-3 protein and dense mononuclear cellular infiltration (100×). **c**: section of liver from the *AV*-treated group showing negative staining and hence negative expression to the caspase-3 protein (100×). **d**: moderate expression of caspase-3 was shown in the hepatocytes of the Cd + *AV*-treated group (100×)

Results of the activated caspase-3 protein expression in the liver of the Cd-intoxicated group revealed an increase in the caspase-3 expression (Fig. 7b), as compared to the control group (Fig. 7a), confirming cellular death. Controversy, *AV* gel ingestion along with Cd mitigates this upregulation (Fig. 7d). Notably, no active caspase-3 protein expression was found in either the control standard group or the *AV*-treated groups (Fig. 7c).

### Impacts of *AV* Gel and Cd on COL-1 and MMP-9

**Fig. 8:**
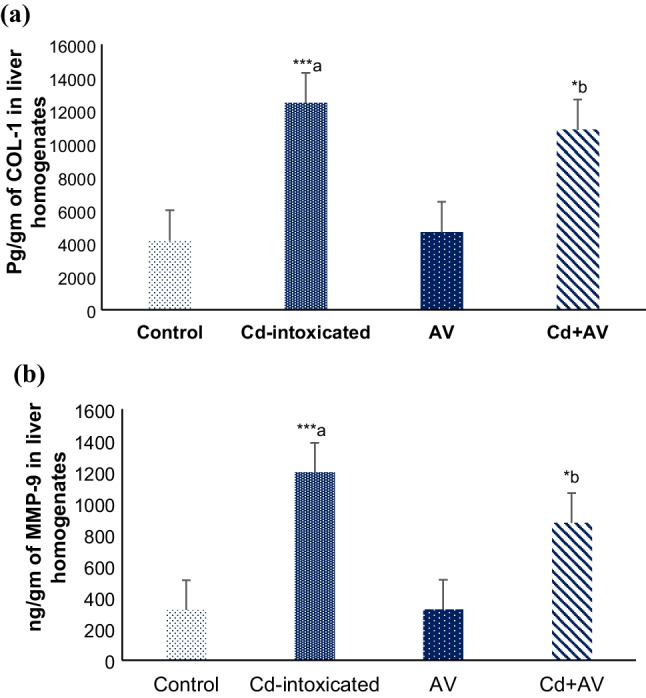
Changes in concentrations of COL-1 (**a**) and MMP-9 (**b**) following Cd and *AV* gel ingestion. Results are given as mean ± SE (*n*=10). ^***a^*p* ≤ 0.001 when compared to control group and ^*b^*p ≤* 0.05 when compared to Cd-ingested group

The COL-1 and MMP-9 parameters were chosen to evaluate the hepatotoxic effect of Cd and the modulatory impact of *AV* gel on the liver microenvironment. Results revealed a highly significant increase (*p* ≤ 0.001) in both COL-1 (Fig. 8a) and MMP-9 (Fig. 8b) concentrations in the Cd-ingested group’s liver homogenate, compared to the healthy normal control. However, the rise in both parameters was considerably (*p* ≤ 0.05) reduced in Cd + AV-treated group to an acceptable level, in comparison with the Cd-ingested rats. Collectively, when compared to the conventional control group, the *AV* group showed no significant changes in COL-1 or MMP-9 concentrations.

## Discussion

Cd has been grouped as an environmental pollutant in most hazardous chemicals list by the World Health Organization (WHO) and the US Agency for Toxic Substances and Disease Registry (ATSDR). At initial stages of Cd exposure, hepatocellular damage has been reported to be greater than renal injury [21, 22]. Thus, searching for palliative therapy for preventing Cd-induced hepatic injury has become crucial nowadays. This study sheds light on scrutinizing and developing an efficient herbal-based hepatoprotective agent against Cd-induced injury following oral exposure to cadmium chloride. *Aloe vera* gel was chosen for this purpose because of its various active components including polysaccharides, anthraquinone, lectin, superoxide dismutase, glycoprotein, vitamins C and E, salicylic acids, and amino acids [23], allowing us to explore its impact on accomplishing this goal. In adult human, sufficient proofs are reported for Cd-triggered Kupffer cell activation, neutrophil infiltration, necrotic hepatocellular death, non-alcoholic fatty liver disease (NAFLD), and non-alcoholic steatohepatitis (NASH) [24].

In the current study, higher levels of Cd residues (1.45 ± 0.05) were detected in liver homogenate of Cd-ingested rats, after three consecutive weeks of oral ingestion with a dose of 3 mg/kg b.w. These levels were significant (*p* ≤ 0.05), compared with unexposed animals (1.1 ± 0.09). Upon absorption, Cd is carried throughout the body and generally attached to a protein with a sulfhydryl group, such as metallothionein (MT). Cd is difficult to be biodegraded via hepatic enzymes and unfortunately, like most heavy metals, tends to be bioaccumulated with the risk of liver injury. Moreover, excretion of Cd and its chemical derivatives is challenging. This is due to its minimal excretion rate via urine and its difficulty via biliary excretion because of the rapid uptake from the intestine back to the liver via the entero-hepatic circulation **[**25**–**27**]**. *AV* gel demonstrates metal chelating ability and has been proven to decrease the absorption of Cd from the GIT due to its enrichment with polyphenols such as tanins, saponins, and flavonoids [28–30]. *AV* gel contains more than 75 different active constituents, including minerals such as zinc, copper, selenium, and calcium. Selenium has been known to counteract the toxicity of heavy metals such as Cd [31], which clarifies the significant reduction in hepatic Cd levels detected in the *AV* gel-ingested rats.

The hepatotoxic impact of Cd has been linked to free radical-mediated oxidative stress, lipid and protein peroxidation, inflammation as well as apoptosis. Cd also reduces the antioxidant defense, jeopardizing the hepatocytes to oxidative damage [22, 32, 33]. According to Teschke (2022) [34], acute Cd liver injury can be triggered either by binding of Cd^2+^ to sulfhydryl groups causing mitochondrial oxidative stress and direct hepatocellular injury based on mitochondrial functional impairment or by Kupffer cell activation with the implication of inflammatory and cytotoxic mediators, such as cytokines and chemokines. Collectively, Cd-induced oxidative stress was ascribed to bioaccumulation and ROS generation [34]. Our data indicated that Cd ingestion significantly (*p* ≤ 0.001) evoked the production of double NO radical content (89.9 ± 0.28) in liver tissue of Cd-treated rats, versus the untreated animals (46.5 ± 0.23). The observed phenomenon could be described by the fact that Cd increases inducible nitric oxide synthase 2 (iNOS2) enzyme activity in the liver, which in turn produces a high level of NO radical. NO interacts with oxygen radicals generating peroxynitrite, a powerful oxidative and nitrosative agent that causes damage to liver by directly affecting cellular macromolecules [35]. Consequently, significant elevation in the MDA levels (13.07 ± 0.14; *p* ≤ 0.001) were also detected in the serum of rats following Cd exposure, compared to the unexposed ones (6.37 ± 0.17). MDA is the most notorious player of lipid peroxidation whose maliferous activities result in parenchymal cell injury [22]. It binds to various cellular components including DNA, advanced glycation end products (AGEs), and acetaldehyde, producing adducts that, in turn, damage the cellular integrity [36].

Paroxanase (PON1) is an antioxidant enzyme with lactonase and esterase activity. It is largely produced within the liver and circulates in association with plasma high-density lipoproteins (HDL), contributing to its ability to reduce low density lipoprotein (LDL) and hydrolyze lipid peroxides [37]. PON1 has been hypothesized to regulate hepatic parenchymal cell death, and hence its overexpression protects against the development of experimental liver disease. Indeed, the link between low PON1 levels and increased susceptibility to the development of liver damage has been well established [38]. In the current study, exposing rats to Cd obviously decreased serum PON1 concentration (227.8 ± 1.4; *p* ≤ 0.05) versus the control group (333.3±1.8), demonstrating the increase in ROS generation and illustrating the lipid peroxidation state induced by Cd intoxication, thus, confirming its ability to trigger liver damage [39].

Heat shock proteins (HSPs) are expressed by cells as an anti-injury mechanism of hepatic tissues. As a result, changes in HSPS levels account as good biomarkers for heavy metals-induced oxidative stress [40]. The HSP70 family was chosen in this study to assess the modulatory effect of *AV* gel on Cd-induced hepatotoxicity since it has previously been postulated that the HSP70 family is more vulnerable to Cd poisoning than other HSPs. Our findings revealed a marked increase in the serum HSP70 levels (7.9 ± 0.52; *p* ≤ 0.05) of Cd-administrated rats, compared to the standard control animals (5.0 ± 0.4). Accordingly, Akiyama et al. [41] previously confirmed Cd-induced hepatotoxicity and demonstrated an increase in HSP70 concentration in primary mouse hepatocytes.

Fortunately, *AV* gel with its valuable content of antioxidants like vitamin C, vitamin E, and other anthraquinone compounds as well as polysaccharides provided remarkable protection and recovery of liver function by modulating Cd-evoked oxidative stress in experimental animals, as evidenced by a notable reduction of hepatic NO radical (75.03 ± 0.14; *p* ≤ 0.001), serum MDA (9.13 ± 0.18; *p* ≤ 0.001), and HSP70 (6.85 ± 0.13; *p* ≤ 0.05) levels with a significant restoration of serum PON1 levels (274.9 ± 1.5; *p* ≤ 0.05) , compared to the Cd-intoxicated group. Indeed, these antioxidants combinations in the *AV* gel are greatly due to anthraquinones (aloin and emodin) and other related compounds, which potentiate its peroxyl radical scavenger activity and augment its protective effects against Cd-induced toxicity in liver and other tissues [42].

As mentioned previously, Cd possessed hepatotoxicity through two routes; the primary one involves initial harm caused by the metal’s direct actions and ROS release and the other for the following injury caused by inflammation. Substantial evidence regards Cd exposure could impact the inherent regulation of Nrf2 and NF-κB signaling pathways to cause oxidative injury in different tissues [43, 44]. Oxidative stress could provoke many pro-inflammatory processes by a series of upstream cellular signals, especially NF-κB pathway [45, 46].

After being exposed to Cd, activated Kupffer cells initiate signaling cascades involving CD14, MyD88, MD-2, mitogen-activated protein kinases (c-Jun N-terminal kinase (JNK)), and NF-κB [47]. NF-κB was shown to activate genes inside the nucleus, which are involved in the regulation of oxidative stress, inflammatory response, and apoptosis [48]. Our data presented a significant upregulation of the NF-κB P65 gene in Cd-intoxicated rats compared to their normal counterparts. Consequently, a significant augmentation of serum IL-6 was also detected in the same group following Cd ingestion. Similarly, He et al. [49] noticed a marked increase in the hepatic protein expression levels of NF-κB P65 (sixfold) and mRNA expression levels of IL-6 and IL-1β, the master regulators of fibrosis by about 2.6 and 6.6-fold, respectively, in CdCl_2_-treated mice versus normal untreated animals, which suggest suffering of liver inflammation.

On the contrary, combining *AV* gel with CdCl_2_ protected against inflammation by lowering NF-κB p65 relative mRNA expression and IL-6 levels. *AV* possesses organ-protective activity, particularly for the liver [50]. Kaempferol, a flavonoid component of *AV* gel, suppresses NF-κB activity, NF-kB-DNA interaction, and nuclear translocation of NF-kB p65 [51], thus inhibiting the progress of inflammatory and apoptotic pathways. Furthermore, it was discovered that zinc (one of the minerals present in *AV* gel) inhibits inflammation and hepatocyte death by lowering interleukin IL-1 and IL-6 mRNA expression levels [52, 53]. Additionally, the anti-inflammatory activity of *AV* gel was attributed to the significant amounts of the polysaccharide gel acemannan, aloe-emodin, and aloin [54]. Additionally, the glucomannan ingredient in *AV* gel, which is one of the polysaccharides rich in mannose, and gibberellin have an anti-inflammatory function via regulating the NF-κB pathway [55].

Thus, Cd exposure triggers the immune cells to secrete pro-inflammatory and pro-fibrogenic factors which activate dormant HSCs, resulting in an excessive ECM accumulation and loss of liver architecture and function [56, 57]. Activated HSCs are primarily responsible for increased ECM deposition by producing an excessive amount of ECM components, including COL-1 [58].

MMPs are a zinc-dependent enzymes family that degrade ECM and act as a marker of HSCs activation**.** Geervliet and Bansal [58] found highly significant increase in MMP-9 expression in acute liver failure. In the current work, oral intake of Cd for three consecutive weeks significantly induced (*p* ≤ 0.001) the production of more MMP9 in hepatic tissues, indicating liver destruction. Lian et al. [59] proved that Cd induced MMP-9 expression via NADPH oxidase/ROS-dependent EGFR/Akt/NF-kB and EGFR/MAPKs (Erk1/2, JNK1/2)/AP-1 axis in human endothelial ECV304 cells.

Additionally, in our study, apoptosis was confirmed in the Cd-intoxicated group by both immunostaining for the cleaved caspase-3 protein expression and by the molecular assessment of both apoptotic and antiapoptotic factors, Bax and Bcl2 respectively. Generated ROS due to Cd toxicity led to protein misfolding, proteotoxic insults, apoptosis, cell dysfunction, and protein aggregation [60–62]. ROS serves as common intracellular mediators of NF-κB activation and can therefore trigger both apoptotic and necrotic cell death depending on the severity of the oxidative stress [63]. Moreover, accumulated Cd has shown to contribute in the development of ferroptosis, a novel mode of cell death discovered in the past few years [64]. This new type of cell death is mainly activated by ROS and caused by the imbalance between the production and breakdown of lipid reactive species in the cell. Ferroptosis can act either directly or indirectly on glutathione peroxidase (GPXs) through various pathways, leading to accumulation of ROS, decreased cell antioxidant capacity, and finally cell oxidative death [65]. Additionally, increased levels of ROS were shown to trigger also pyroptosis. Previous studies confirmed that exposure to Cd induced pyroptosis of neuronal, lymphocyte, as well as vascular endothelial cells by activating the NLRP3 inflammasome complex [66].

Fortunately, treatment with *AV* gel greatly mitigated the apoptotic status of liver cells by significantly modulating the expression levels of both apoptotic and antiapoptotic levels. The dramatic increase in the Bax to Bcl2 ratio (8.34) confirmed the Cd-induced apoptosis in hepatic cells. Controversy, this ratio was notably reduced 4 times, reaching approximately 2.13 following co-administration of *AV* gel, which greatly confirmed the anti-apoptotic effect of *AV* in liver tissues. This anti-apoptotic activity of *AV* gel was collectively due to its antioxidant and anti-inflammatory effects. Moreover, anti-fibrotic activity *AV g*el against Cd-induced liver injury was improved by the significant reduction in the levels of COL1 and MMP-9. This activity is attributed to the synergistic effect of the valuable flavonoids such as quercetin, naringenin, and kaempferol which possess antioxidant, anti-inflammatory, and anti-apoptotic activities [67–70]. It is noteworthy that *AV* was enriched with Aloe-emodin, a potent naturally anthraquinone ingredient which exhibited hepatoprotective effect by treating various diseases including liver fibrosis [71].

In conclusion, acute ingestion of CdCl_2_ by rats led to bioaccumulation of Cd in the liver, which in turn triggered oxidative impulse, inflammation, and apoptosis causing hepatic damage. Our data declared the efficiency of *AV* gel with its valuable constituents in protecting liver cells against Cd toxicity via attenuating oxidative impacts, lowering inflammation, and inhibiting apoptosis. These findings suggested that *AV* gel could be approved as a promising hepatocellular protective agent against hepatocellular damage due to Cd exposure.

## Data Availability

All data and materials are fully available within the manuscript.
